# Spermine: an endogenous iron chelator that suppresses ferroptosis

**DOI:** 10.1186/s43556-026-00614-1

**Published:** 2026-10-02

**Authors:** Yutian Xia

**Affiliations:** Faculty of Health, Medicine and Behavioural Sciences, Frazer Institute, The University of Queensland, Brisbane, QLD 4102 Australia

A recent study published in *Nature* by Li and colleagues identified spermine, a ubiquitous polyamine, as an endogenous Fe^2+^ chelator that directly suppresses ferroptosis [1]. In hepatocellular carcinoma (HCC), the study established an aldehyde dehydrogenase 18 family member A1 (ALDH18A1)-dependent pathway for de novo spermine synthesis that reduces labile Fe^2+^ availability and limits lipid peroxidation. In ischaemia–reperfusion injury (IRI) models, ALDH18A1 remained unchanged; instead, increased polyamine catabolism depleted endogenous spermine, whereas exogenous spermine reduced labile Fe^2+^ accumulation, lipid peroxidation and tissue injury. Together, these findings suggest that spermine-mediated Fe^2^⁺ chelation influences ferroptosis susceptibility in a context-dependent manner.

The study first documents a biphasic pattern of ferroptosis during hepatocarcinogenesis in two established mouse models. Early lesions exhibit elevated lipid peroxidation and labile Fe^2+^, consistent with ferroptotic pressure that constrains tumour development, whereas advanced tumours show reduced ferroptosis markers despite increased total iron content (Fig. 1). This temporal pattern suggests that emerging tumour cells progressively acquire metabolic defences against an initial ferroptotic vulnerability. Untargeted metabolomics and flux analyses revealed consistent upregulation of the polyamine biosynthetic pathway in HCC, with spermine and its intermediates ranking among the most elevated metabolites. Although polyamine catabolism can generate pro-ferroptotic species such as H_2_O_2_ and acrolein, the study identifies a direct ferroptosis-suppressive role for intact spermine, independent of its catabolic products.

**Fig. 1:**
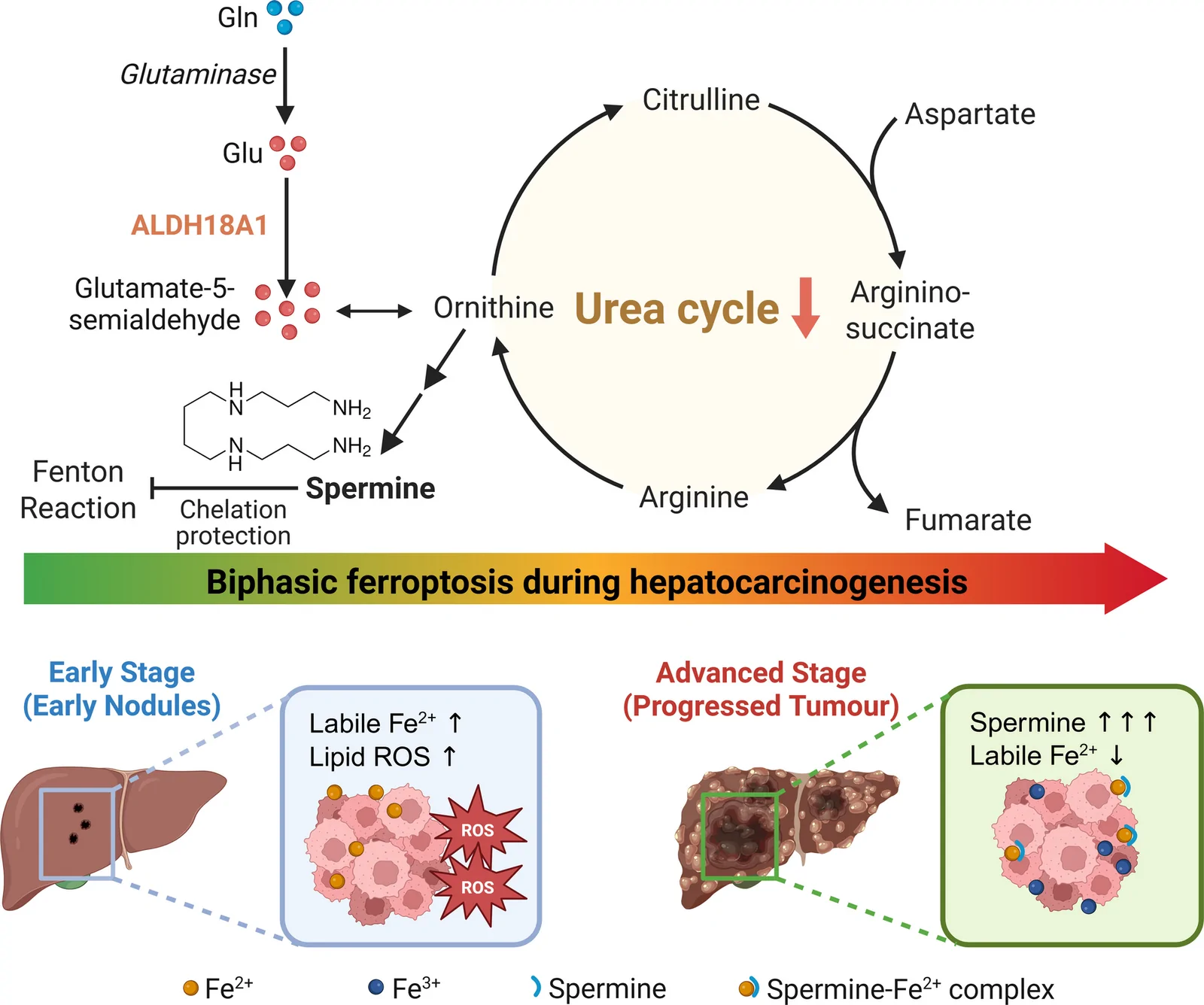
Schematic representation of the ALDH18A1-spermine axis during hepatocarcinogenesis. Suppression of the urea cycle in HCC channels glutamine metabolism through the ALDH18A1-dependent glutamate-P5C-ornithine pathway to support de novo spermine synthesis. Spermine coordinates labile Fe^2+^ through Fe–N interactions with near 1:1 stoichiometry, forming a redox-inactive complex and reducing the Fe^2+^ available for Fenton chemistry (Fe^2+^  + H_2_O_2_ → Fe^3+^  + OH^−^ + •OH). During early hepatocarcinogenesis, increased labile Fe^2+^ and lipid reactive oxygen species impose ferroptotic pressure, whereas advanced tumours accumulate spermine and show reduced labile Fe^2+^, thereby limiting lipid peroxidation and ferroptosis. Upward and downward arrows indicate increased and decreased abundance respectively. (Created with BioRender.com)

In HCC, suppression of the urea cycle is accompanied by ALDH18A1-dependent channelling of glutamine metabolism through the glutamate-pyrroline-5-carboxylate (P5C)-ornithine pathway, thereby supporting ornithine production and subsequent de novo spermine synthesis. This metabolic rewiring maintains spermine abundance, reduces the cellular labile Fe^2+^ pool and limits ferroptosis under chronic oxidative stress (Fig. 1). Genetic deletion or AAV-mediated knockdown of *ALDH18A1*, as well as pharmacological inhibition with YG1702 (an ALDH18A1 inhibitor), consistently depleted spermine, increased labile Fe^2+^ and lipid peroxidation, and suppressed both spontaneous and chemically induced hepatocarcinogenesis. In cellular rescue experiments, exogenous spermine restored spermine abundance and reversed the increases in labile Fe^2+^ and lipid peroxidation caused by ALDH18A1 loss. In mouse HCC models, spermine and liproxstatin-1 (Lip-1) reduced malondialdehyde accumulation and reversed the reduction in tumour formation caused by *ALDH18A1* knockdown or inhibition. In contrast, Lip-1 acted downstream by suppressing ferroptosis-associated lipid peroxidation and did not restore spermine abundance.

Multiple biophysical and functional approaches converged to support spermine as a direct Fe^2+^ chelator. An NBD-labelled spermine analogue colocalized with labile Fe^2+^ detected by FerroOrange, and isothermal titration calorimetry showed an interaction with near 1:1 stoichiometry and a micromolar dissociation constant. Spectroscopic analyses further supported Fe–N coordination and formation of a redox-inactive complex. Importantly, the two cell-free assays addressed distinct questions. In the FerroOrange assay, deferoxamine was the comparator for Fe^2+^ chelation, complex formation and redox behaviour. In the separate reconstituted liposomal assay, spermine inhibited Fe^2+^-driven lipid peroxidation with an efficacy comparable to ferrostatin-1, whereas putrescine and spermidine showed little activity. These data support the description of spermine as an endogenous metabolic iron buffer. Independent work by Sharma and colleagues showed that polyamines can coordinate Fe^2+^ and that their depletion expands the labile iron pool and increases sensitivity to GPX4 inhibition in K562 and other cellular models [2]. Simultaneous measurements using genetically encoded polyamine and redox-active iron reporters revealed a strong inverse relationship between the two metabolic pools at single-cell resolution [2]. Collectively, these findings support a broader polyamine-mediated iron-buffering mechanism across diverse cellular contexts, whereas the ALDH18A1-dependent spermine biosynthetic pathway has been specifically demonstrated in HCC. Beyond iron chelation, polyamine metabolism has also been linked to immune regulation through cGAMP-STING, JAK and cGAS signalling [3–5]. These observations raise the possibility that polyamine metabolism intersects with ferroptosis resistance and immune regulation, although direct crosstalk with spermine-Fe^2+^ chelation has not been demonstrated.

Stable isotope tracing with ^15^N-labelled glutamine and methionine demonstrated increased incorporation of these precursors into polyamines in two mouse models of HCC relative to non-malignant controls. Combined with the genetic and pharmacological evidence, these findings support a role for ALDH18A1-dependent spermine synthesis in protecting HCC cells from ferroptosis. Tumours with pronounced urea-cycle defects may therefore be particularly sensitive to ALDH18A1 inhibition. However, because ALDH18A1 also contributes to proline biosynthesis, metabolic compensation and collateral effects during sustained inhibition will need to be defined before this strategy can be translated.

The authors further evaluated the role of spermine-mediated Fe^2+^ chelation in ischaemia–reperfusion injury (IRI). In these models, ALDH18A1 abundance remained unchanged, whereas increased polyamine catabolism was associated with endogenous spermine depletion. In mouse models of hepatic, intestinal and renal IRI, spermine administration initiated before ischaemia reduced labile Fe^2+^ accumulation, lipid peroxidation and organ injury; the renal protocol included an additional post-reperfusion dose. By limiting labile iron upstream of Fenton chemistry, spermine could offer a mode of protection mechanistically distinct from direct radical scavenging. However, the available data do not establish superior or more durable protection in IRI relative to radical-scavenging ferroptosis inhibitors; dedicated comparative studies with extended follow-up are needed. The two disease contexts suggest opposing interventions: ALDH18A1 inhibition in HCC and timed spermine supplementation in IRI. Both approaches will require careful optimization of dose, timing, delivery and tissue selectivity.

Several mechanistic and translational questions nevertheless remain. Spermine was tested at 10 µM, a concentration described as approximating physiological extracellular levels, and its measured dissociation constant for Fe^2+^ was in the micromolar range. Moreover, tissue spermine concentrations exceeded measured Fe^2+^ levels by more than 20-fold in the IRI models. These observations are consistent with a physiologically relevant role for spermine-mediated Fe^2+^ chelation, but the availability, distribution and binding occupancy of spermine and Fe^2+^ within specific cellular compartments remain unclear. Direct tracking of the relevant iron pools and spermine-Fe^2+^ complexes will be needed to determine where and when this interaction is functionally important. Competition with ferritin and other intracellular iron-binding molecules, as well as the fate of the resulting complexes, also requires investigation. ALDH18A1 overexpression mitigated ferroptosis after ferroptosis suppressor protein 1 (FSP1) deletion, suggesting that ALDH18A1-mediated protection can persist in the absence of FSP1; however, the mechanistic relationship between these defence pathways remains unclear. Future work should determine whether selective ALDH18A1 inhibition can induce tumour ferroptosis in vivo and tissue-targeted spermine delivery can provide peri-ischaemic protection without compromising systemic polyamine balance or iron homeostasis.

The identification of spermine as an endogenous Fe^2+^ chelator extends current understanding of ferroptosis defence mechanisms and highlights metabolite-mediated iron buffering as an additional mode of ferroptosis regulation. Importantly, the study shows that changes in spermine abundance can directly influence the cellular labile Fe^2+^ pool and ferroptosis susceptibility in specific disease settings. The therapeutic implications appear to be context dependent: inhibiting ALDH18A1-dependent spermine synthesis may expose a metabolic vulnerability in HCC, whereas appropriately timed spermine supplementation may protect tissues from peri-ischaemic injury.

## Data Availability

Not applicable.

## References

[1] Li M, Yu X, Ouyang S, Chen X, Yu H, Liu Y, et al. Spermine is an endogenous iron chelator that inhibits ferroptosis. Nature. 2026;655(8121):240–50. 10.1038/s41586-026-10597-2. 42236947

[2] Sharma P, Keys HR, Mansell RP, Stark J, Girard L, Ausler C, et al. Polyamines buffer labile iron to suppress ferroptosis. Cell. 2026;189(18):5571-89.e10. 10.1016/j.cell.2026.07.040. PMC13527661 42600612

[3] Ma Y, Zhao C, Guo J, Fu Y, Wang W, Zhang J, et al. Polyamine sequestration of 2′3′-cGAMP constrains intercellular transmission and STING engagement to subvert antitumor immunity. J Clin Invest. 2026;136(11):e201460. 10.1172/JCI201460. PMC13221228 42222888

[4] Xu H, Zhang X, Wang X, Li B, Yu H, Quan Y, et al. Cellular spermine targets JAK signaling to restrain cytokine-mediated autoimmunity. Immunity. 2024;57(8):1796-811.e8. 10.1016/j.immuni.2024.05.025. 38908373

[5] Zhao C, Ma Y, Zhang M, Gao X, Liang W, Qin Y, et al. Polyamine metabolism controls B-to-Z DNA transition to orchestrate DNA sensor cGAS activity. Immunity. 2023;56(11):2508-22.e6. 10.1016/j.immuni.2023.09.012. 37848037

